# DOES DISTANCE CARE MAKE A DIFFERENCE IN THE STATUS OF CARE RECIPIENTS OVER AN 18-MONTH PERIOD?

**DOI:** 10.1093/geroni/igac059.661

**Published:** 2022-12-20

**Authors:** Tomoko Wakui, Ichiro Kai, Shuichi Awata

**Affiliations:** Tokyo Metropolitan Institute of Gerontology, Tokyo, Tokyo, Japan; The University of Tokyo, Tokyo, Tokyo, Japan; Tokyo Metropolitan Institute of Gerontology, Tokyo, Tokyo, Japan

## Abstract

Aging in place is a priority for policy makers and individuals, and the continuity of care provided at home is one of the major concerns. Co-resident caregiving has been a dominant caregiving arrangement in Japan; however, the solitary residence of older care recipients has become more frequent. This study thus examined the impact of distance care on the status of care recipients over an 18-month period. Longitudinal data were collected from family caregivers of older adults in 17 municipalities of north-central Japan beginning in 2010. The status of care recipients after 18 months was categorized as receiving care at home, being institutionalized, deceased, hospitalized, moved out from the community, recovered, or untraceable. Care recipients’ age, gender, activities of daily living, dementia symptoms, and caregivers’ age, gender, health status, economic status, and caregiving burden were measured. We analyzed data of 2280 care recipients–caregiver dyads. Care recipients’ mean age was 84.7 (SD=7.3), and 66.7% were women. Of those, 65.0% had some dementia symptoms. Multivariate logistic regression models predicting the status of care recipients after 18 months showed that distance care had significant effects on care recipients’ status. Those who received distance care were more likely to be moved out, institutionalized, and deceased after 18 months, even after controlling for their care needs and caregiving situations. There were no significant effects on being hospitalized. This study suggests that the more difficult situation pertaining to staying at home is experienced by care recipients who received distance care, and support is required.

